# A formal concept analysis approach to consensus clustering of multi-experiment expression data

**DOI:** 10.1186/1471-2105-15-151

**Published:** 2014-05-19

**Authors:** Anna Hristoskova, Veselka Boeva, Elena Tsiporkova

**Affiliations:** 1 Department of Information Technology, Ghent University - iMinds, Gaston Crommenlaan 8 (201), 9050 Ghent, Belgium; 2 Department of Computer Systems and Technology, Technical University of Sofia-branch Plovdiv, Tsanko Dyustabanov 25, 4000 Plovdiv, Bulgaria; 3 ICT & Software Engineering Group, Sirris, The Collective Center for the Belgian Technological Industry, Reyerslaan 80, 1030 Brussels, Belgium

**Keywords:** Consensus clustering, Particle swarm optimization, Formal concept analysis, Integration analysis, Multi-experiment expression data

## Abstract

**Background:**

Presently, with the increasing number and complexity of available gene expression datasets, the combination of data from multiple microarray studies addressing a similar biological question is gaining importance. The analysis and integration of multiple datasets are expected to yield more reliable and robust results since they are based on a larger number of samples and the effects of the individual study-specific biases are diminished. This is supported by recent studies suggesting that important biological signals are often preserved or enhanced by multiple experiments. An approach to combining data from different experiments is the aggregation of their clusterings into a consensus or representative clustering solution which increases the confidence in the common features of all the datasets and reveals the important differences among them.

**Results:**

We propose a novel generic consensus clustering technique that applies Formal Concept Analysis (FCA) approach for the consolidation and analysis of clustering solutions derived from several microarray datasets. These datasets are initially divided into groups of related experiments with respect to a predefined criterion. Subsequently, a consensus clustering algorithm is applied to each group resulting in a clustering solution per group.

These solutions are pooled together and further analysed by employing FCA which allows extracting valuable insights from the data and generating a gene partition over all the experiments. In order to validate the FCA-enhanced approach two consensus clustering algorithms are adapted to incorporate the FCA analysis. Their performance is evaluated on gene expression data from multi-experiment study examining the global cell-cycle control of fission yeast. The FCA results derived from both methods demonstrate that, although both algorithms optimize different clustering characteristics, FCA is able to overcome and diminish these differences and preserve some relevant biological signals.

**Conclusions:**

The proposed FCA-enhanced consensus clustering technique is a general approach to the combination of clustering algorithms with FCA for deriving clustering solutions from multiple gene expression matrices. The experimental results presented herein demonstrate that it is a robust data integration technique able to produce good quality clustering solution that is representative for the whole set of expression matrices.

## Background

DNA microarray technology offers the ability to screen the expression levels of thousands of genes in parallel under different experimental conditions or their evolution in discrete time points. All these measurements contain information on several aspects of gene regulation and function, ranging from understanding the global cell-cycle control of microorganisms [1], to cancer in humans [2,3]. Gene clustering is one of the most frequently used analysis methods for gene expression data. Clustering algorithms are used to divide genes into groups according to the degree of their expression similarity. These groups suggest the correlation and/or co-regulation of the respective genes that possibly share common biological roles.

The combination of data from multiple microarray studies addressing a similar biological question is gaining high importance in the recent years [4-7] due to the ever increasing number and complexity of the available gene expression datasets. The integration and evaluation of multiple datasets yield more reliable and robust results since they are based on a larger number of samples and the effects of the individual study-specific biases are diminished. A method for integration analysis of the data from multiple experiments is the aggregation of their clustering results into a consensus clustering which emphasizes the common organization in all the datasets and reveals the significant differences among them.

In this work, we present and validate a novel generic approach to consensus clustering based on Formal Concept Analysis (FCA) where microarray data realized under different experimental conditions is integrated into a representative consensus clustering solution. It initially divides the available microarray experiments into groups of related datasets with respect to a predefined criterion and then a consensus clustering algorithm is applied to each group of experiments separately. The rationale behind this is that if the experiments are closely related to one another, then they produce more accurate and robust clustering solution. Next, the clustering solutions produced by the different groups are pooled together and further analyzed by employing FCA which allows extracting valuable insights from the data and generating a gene partition over the whole experimental compendium. FCA produces a concept lattice where each concept represents a subset of genes that belongs to a number of clusters. The concepts compose the final disjoint clustering partition.

The proposed general *FCA-enhanced* consensus clustering approach is experimentally validated. For this purpose, two consensus clustering methods are adapted to incorporate an FCA analysis step. These methods are quite different; the first (Integrative) integrates the partitioning results derived from multiple microarray datasets through a weighted aggregation process, while the second employs a Particle Swarm Optimization (PSO) approach to cluster gene expression data across multiple experiments. The FCA results derived from both methods are analysed with respect to the cluster consistency and biological relevance. It is shown that although both algorithms optimize different clustering characteristics, FCA is able to construct similar consensus clustering solutions that are representative for the whole set of experiments.

In summary, the main contribution of the introduced *FCA-enhanced* consensus clustering technique is that it proposes a general approach to the combination of clustering algorithms with Formal Concept Analysis (FCA) for deriving clustering solutions from multiple gene expression matrices. In addition, the approach is demonstrated to be independent of the selected clustering algorithm. In this way one can use a customized algorithm optimized for the specific characteristics of each group of experiments. The further employment of FCA allows performing a subsequent data analysis, which provides useful insights on the biological role of genes contained in the same FCA concepts.

## Related work

Presently, with the increasing number and complexity of available gene expression datasets, the combination of data from multiple microarray studies addressing a similar biological question is gaining importance. However, as emphasized in [7], the investigations of gene expression levels have also generated controversy because of the probabilistic nature of the conclusions and the discrepancies between the results of the studies addressing the same biological question. Subsequently, the authors proposed data analysis and visualization tools for estimating the degree to which the findings of one study are reproduced by others and for integrating multiple studies in a single analysis. These tools were described in the context of studies of breast cancer and it was illustrated that it is possible to identify a substantial biologically relevant subset of the human genome within which the expression levels are reliable. The latter suggests that important biological signals are often preserved or enhanced by multiple experiments.

Another approach to combining data from different experiments is the aggregation of their clusterings into a consensus or representative clustering which increases the confidence in the common features in all the datasets and reveals the important differences among them [8]. Methods for the combination of clustering results derived for each dataset separately have been considered in [9-11]. The algorithm proposed in [9] first generates local cluster models and then combines them into a global cluster model of the data. The study in [10] focuses on clustering ensembles, *i.e.* seeking a combination of multiple partitions that provides improved overall clustering of the given data. The combined partition is found as a solution to the corresponding maximum likelihood problem using the Expectation-Maximization (EM) algorithm in [12]. The authors in [11] consider the problem of combining multiple partitions of a set of objects into a single consolidated clustering without accessing the features or algorithms that determined these partitions. The cluster ensemble problem is formalized as a combinatorial optimization problem in terms of shared mutual information.

In contrast to the foregoing approaches, the generic solution proposed in this paper applies FCA in order to construct a consensus clustering that is representative for all the datasets and in addition, it is independent of the applied clustering algorithm. In order to validate the proposed *FCA-enhanced* approach two consensus clustering algorithms have been developed, *Integrative*[13] and *PSO-based*[14], and used in the validation process. Note that, a preliminary *FCA-enhanced* version of the *PSO-based* algorithm was initially considered in [15].

In [13] we study two microarray data integration techniques that can be applied to the problem of deriving clustering results from a set of microarray experiments. A cluster integration approach is considered, which combines the information contained in multiple microarray experiments at the level of expression or distance matrices and then applies a clustering algorithm on the combined matrix. Furthermore, a technique for the combination of partitioning results derived from multiple microarray datasets, referred to as *Integrative consensus clustering*, is introduced. It uses a traditional aggregation schema in order to integrate the different partitioning results into a final partition matrix.

The *PSO-based* approach is used to cluster gene expression data across multiple experiments. In this algorithm, referred to as *PSO-based consensus clustering*, each experiment (dataset) defines a particle which is initialized with a set of *k* cluster centroids obtained after performing k-means clustering on the experiment. The final (optimal) clustering solution is found by updating the particles using the information on the best clustering solution obtained by each experiment and the entire set of experiments.

In this article, the *Integrative* and *PSO-based* consensus clustering approaches are extended incorporating a final FCA-step. The experimental results from their validation suggest that although both algorithms optimize different clustering characteristics, FCA is able to construct similar consensus clustering solutions.

## Methods

### Partitioning algorithms

Three partitioning algorithms are commonly used for the purpose of dividing data objects into *k* disjoint clusters [16]: k-means, k-medians and k-medoids clustering. All three methods start by initializing a set of *k* cluster centres, where *k* is preliminarily determined. Subsequently, each object of the dataset is assigned to the cluster whose centre is the nearest, and the cluster centres are recomputed. This process is repeated until the objects inside every cluster become as close to the centre as possible and no further object item reassignments take place. The EM algorithm in [12] is commonly used for that purpose, *i.e.* to find the optimal partitioning into *k* groups. The three partitioning methods in question differ in how the cluster centre is defined. In k-means, the cluster centre is defined as the mean data vector averaged over all objects in the cluster. Instead of the mean, k-medians calculates the median for each dimension in the data vector. Finally, in k-medoids [17], which is a robust version of the k-means, the cluster centre is defined as the object which has the smallest sum of distances to the other objects in the cluster, *i.e.*, this is the most centrally located point in a given cluster.

### Particle swarm optimization

*Particle swarm optimization* (PSO) is an evolutionary computation method introduced in [18]. In order to find an optimal or near-optimal solution to the problem, PSO updates the current generation of particles (each particle is a candidate solution to the problem) using the information on the best solution obtained by each particle and the entire population.

The hybrid algorithm proposed in [14] combines k-means and PSO for deriving a clustering result from a group of *n* related microarray datasets *M*_1_,*M*_2_,…,*M*_*n*_. Each dataset contains the gene expression levels of *m* genes in *n*_*i*_ different experimental conditions or time points. In this context, each matrix *i* is used to generate *k* cluster centers, which are considered to represent a particle, *i.e.* the particle is treated as a set of points in an *n*_*i*_-dimensional space. The final (optimal) clustering solution is found by updating the particles using the information on the best clustering solution obtained by each data matrix and the entire set of matrices.

Assume that the *i*-th particle is initialized with a set of *k* cluster centers^a^$C_{i}=\left\{C_{1}^{i},C_{2}^{i},\ldots ,C_{k}^{i}\right\}$ and a set of velocity vectors^b^$V_{i}=\left\{V_{1}^{i},V_{2}^{i},\ldots ,V_{k}^{i}\right\}$ using gene expression matrix *M*_*i*_. Thus each cluster center is a vector $C_{j}^{i}=\left(c_{j1}^{i},c_{j2}^{i},\ldots ,c_{jn_{i}}^{i}\right)$ and each velocity vector is a vector $V_{j}^{i}=\left(v_{j1}^{i},v_{j2}^{i},\ldots ,v_{jn_{i}}^{i}\right)$, *i.e.* each particle *i* is a matrix (or a set of points) in the *k*×*n*_*i*_ dimensional space.

Next, assume that *P*_*g*_={*P*_*g*1_,*P*_*g*2_,…,*P*_*g* *k*_} is a set of cluster centers in an *n*_*g*_-dimensional space representing the best clustering solution found so far within the set of matrices and $P_{i}=\left\{P_{1}^{i},P_{2}^{i},\ldots ,P_{k}^{i}\right\}$ is the set of centroids of the best solution discovered so far by the corresponding matrix. The update equation for the *d*-th dimension of the *j*-the velocity vector of the *i*-th particle is defined as follows

(1) $$\;v_{\text{jd}}^{i}(t\;+\;1)=w\cdot v_{\text{jd}}^{i}(t)\;+\;c_{1}\cdot \varphi_{1}\cdot \left(p_{\text{jd}}^{i}-c_{\text{jd}}^{i}(t)\right)\;+\;c_{2}\cdot \varphi_{2}\cdot g(t),$$

where *i*=1,…,*n*; *j*=1,…,*k*; *d*=1,…,*n*_*i*_ and

(2) $$g(t)=\left\{\begin{array}{l} p_{\text{gd}}-c_{\text{jd}}^{i}(t),\text{if}\;n_{g}\geq n_{i}\\ 0,\text{otherwise} \end{array}\right)$$

The variables *φ*_1_ and *φ*_2_ are uniformly generated random numbers in the range [0,1], *c*_1_ and *c*_2_ are called acceleration constants whereas *w* is called inertia weight as defined in [19]. The first part of Equation (1) represents the *inertia* of the previous velocity, the second part is the *cognition part* that identifies the personal experience of the particle and the third part represents the cooperation among particles and is therefore named the *social component*. Acceleration numbers *c*_1_, *c*_2_ and *inertia* weight *w* are predefined by the user. Note that the *cognition part* in the above equation has a modified interpretation. Namely, it represents the private ‘thinking’ (opinion) of the particle based on its own source of information (dataset). Due to this we adapted the *social part* (see equation (2)) since each particle matrix has a different number of columns (*n*_*i*_) due to different number of experiment points in each dataset. It was demonstrated in [19] that when *w* is in the range [0.9,1.2] PSO will have the best chance to find the global optimum within a reasonable number of iterations. Furthermore, *w*=0.72 and *c*_1_=*c*_2_=1.49 were found in [20] to ensure good convergence.

The clustering algorithm combining PSO and k-means can be summarized as follows: 

1. Initialize each particle with *k* cluster centers obtained as a result of applying the k-means algorithm to the corresponding data matrix.

2. Initialize the personal best clustering solution of each matrix with the corresponding clustering solution found in Step 1.

3. **for** iteration = 1 **to** max-iteration **do**

(a) **for***i*=1**to***n***do** (i.e. for all datasets)

i. **for***j*=1**to***m***do** (i.e. for all genes in the current dataset)

A. Calculate the distance of gene *g*_*j*_ with all cluster centers.

B. Assign *g*_*j*_ to the cluster that has the nearest center to *g*_*j*_.

ii. **end for**

iii. Calculate the fitness function for the clustering solution *C*_*i*_.

iv. Update the personal best clustering solution *P*_*i*_.

(b) **end for**

(c) Find the global best solution *P*_*g*_.

(d) Update the cluster centers according to the velocity updating formula proposed in equation (1).

4. **end for**

The *PSO-based* clustering algorithm was first introduced in [21] showing that it outperforms k-means and a few other state-of-the-art clustering algorithms. In this method, each particle represents a possible set of *k* cluster centroids. The authors in [22] hybridized the approach in [21] with the k-means algorithm for clustering general datasets. A single particle of the swarm is initialized with the result of the k-means algorithm while the rest of the swarm is initialized randomly. In [23] a new approach is proposed based on the combination of PSO and Self Organizing Maps and applied it for clustering gene expression data obtaining promising results. Further the study in [24] considers a dynamic clustering approach based on PSO and genetic algorithm. The main advantage of this algorithm is that it can automatically determine the optimal number of clusters and simultaneously cluster the data set with minimal user interference. The downside of all the foregoing approaches is that they are not suitable for consolidating multiple partitions as the conducted clustering analysis is based on a single expression matrix.

### Formal concept analysis

*Formal Concept Analysis* (FCA) [25] is a mathematical formalism allowing to derive a *concept lattice* from a formal context constituted of a set of objects *O*, a set of attributes *A*, and a binary relation defined as the Cartesian product *O*×*A*. The context is described as a table which rows correspond to objects and the columns to attributes or properties and a cross in a table cell means that “an object possesses a property”. FCA is used for a number of purposes among which knowledge formalization and acquisition, ontology design, and data mining.

The *concept lattice* is composed of formal concepts, or simply concepts, organized into a hierarchy by a partial ordering (a subsumption relation allowing to compare concepts). Intuitively, a concept is a pair (*X*,*Y*) where *X*⊆*O*, *Y*⊆*A*, and *X* is the maximum set of objects sharing the whole set of attributes in *Y* and vice-versa. The set *X* is called the *extent* and the set *Y* the *intent* of the concept (*X*,*Y*). The subsumption (or sub concept - super concept) relation between concepts is defined as follows:

(3) $$\left(X_{1},Y_{1}\right)≺\left(X_{2},Y_{2}\right)\Leftrightarrow X_{1}\subseteq X_{2}\;\left(\text{or}\;Y_{2}\subseteq Y_{1}\right).$$

Relying on this subsumption relation ≺, the set of all concepts extracted from a context is organized within a complete *lattice*. That means that for any set of concepts there is a smallest super concept and a largest sub concept, called the *concept lattice*.

The FCA or *concept lattice approach* has been applied for extracting local patterns from microarray data [26,27] or for performing microarray data comparison [28,29]. For example, the FCA method proposed in [28] builds a *concept lattice* from the experimental data together with additional biological information. Each vertex of the lattice corresponds to a subset of genes that are grouped together according to their expression values and some biological information related to the gene function. It is assumed that the lattice structure of the gene sets might reflect biological relationships in the dataset. In [30], a FCA-based method is proposed for extracting groups or classes of co-expressed genes. A concept lattice is constructed where each concept represents a set of co-expressed genes in a number of situations. A serious drawback of the method is the fact that the expression matrix is transformed into a binary table (the input for the FCA step) which leads to possible introduction of biases or information loss. Thus the authors further propose and compare two FCA-based methods for mining gene expression data and show that they are equivalent [31]. The first one relies on interordinal scaling, encoding all possible intervals of attribute values in a formal context that is processed with classical FCA algorithms. The second one relies on pattern structures without a prior transformation, and is shown to be more computationally efficient and to provide more readable results. Notice that all the mentioned FCA-based methods focus solely on optimizing the clustering of a single expression matrix and consequently, they are not suited for the consolidation of multiple partitions.

### FCA-enhanced consensus clustering algorithm

The problem of deriving clustering results from a set of gene expression matrices can be approached in two different ways: 1) information contained in different datasets may be combined at the level of expression (or similarity) matrices and afterwards clustered; 2) given multiple clustering solutions, one per each dataset, find a consensus (combined) clustering. In this section, a general *FCA-enhanced* consensus clustering algorithm for deriving a clustering result from multiple microarray datasets is proposed which adopts the second approach.

Assume that a particular biological phenomenon is monitored in several high-throughput experiments under *n* different conditions. Each experiment *i* (*i*=1,2,…,*n*) is supposed to measure the gene expression levels of *m*_*i*_ genes in *n*_*i*_ different experimental conditions or time points. Thus, a set of *n* different data matrices *M*_1_,*M*_2_,…,*M*_*n*_ will be produced, one per experiment.

**Figure 1:**
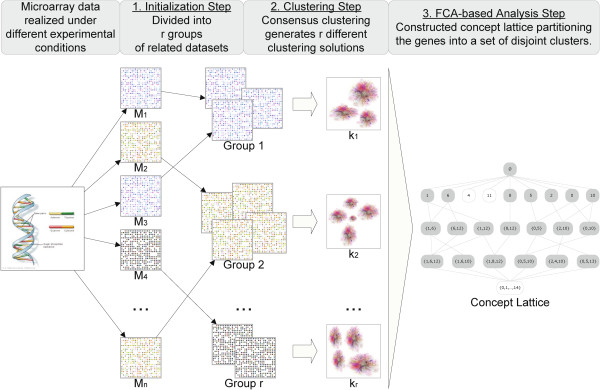
**Schematic representation of the** ***FCA-enhanced* consensus clustering approach.**

The *FCA-enhanced* consensus clustering consists of three distinctive steps in Figure 1: 1) the expression datasets are divided into several smaller groups using some predefined criterion; 2) a consensus clustering algorithm (*e.g.Integrative*, *PSO-based* or other) is applied to each group of datasets separately, which produces a list of different clustering solutions, one per group; 3) these clustering solutions are further transformed into a single clustering result by employing FCA.

In contrast to the consensus clustering algorithms discussed in the foregoing sections, where some partitioning (*e.g.Integrative* and *PSO-clustering*) algorithm is applied to the entire set of experiments in order to produce the final clustering solution, the algorithm proposed herein initially divides the available microarray datasets into groups of related (similar) experiments with respect to a predefined criterion. The rationale behind this is that if the experiments are closely related to one another, then these experiments produce more accurate and robust clustering solution. Thus, the selected consensus clustering algorithm is applied to each group of experiments separately. This produces a list of different clustering solutions, one per each group. Subsequently, these solutions are pooled together and further analyzed by employing FCA which allows extracting valuable insights from the data and generating a gene partition over the whole experimental compendium. FCA produces a *concept lattice* where each concept represents a subset of genes that belong to a number of clusters. The different concepts compose the final disjoint clustering partition.

The proposed *FCA-enhanced* consensus clustering approach has the following characteristics: 

1. the clustering uses all data by allowing potentially each group of related experiments to have a different set of genes, *i.e.* the total set of studied genes is not restricted to those contained into all datasets;

2. it is better tuned to each experimental condition by identifying the initial number of clusters for each group of related experiments separately depending on the number, composition and quality of the gene profiles;

3. the problem with ties is avoided (*i.e.* a case when a gene is randomly assigned to a cluster because it belongs to more than one cluster) by employing FCA in order to analyze together all the partitioning results and find the final clustering solution representative of the whole experimental compendium.

The distinctive steps of the *FCA-enhanced* clustering algorithm, visualized in Figure 1, are explained in detail below.

#### Initialization step

Let us consider the aforementioned expression matrices *M*_1_,*M*_2_,…,*M*_*n*_ monitoring *N* different genes in total. The initialization step is the most variable part of the algorithm since it closely depends on the concrete clustering algorithm employed.

The available gene expression matrices are divided into *r* groups of related (similar) datasets with respect to some predefined criterion, *e.g.* the used synchronized method or the expression similarity between the matrices. For each group the set of studied genes needs to be restricted to those contained in all datasets of the group, *i.e.* the number of overlapping genes found across all datasets of the group. Then the number of cluster centres is identified for each group of experiments *i* (*i*=1,2,…,*r*) separately. As discussed in [32,33], this can be performed by running the k-means or other clustering algorithm on each data matrix for a range of different numbers of clusters. Subsequently, the quality of the obtained clustering solutions needs to be assessed in some way in order to identify the clustering scheme which best fits the datasets in question. Some commonly used validation measures are the Silhouette Index and Connectivity, presented in the Cluster validation measures section, which are able to identify the best clustering scheme. Finally, the prevailing number of clusters within the concrete group of experiments is selected as representative for the whole group.

#### Clustering step

The selected consensus clustering (*e.g.Integrative*, *PSO-based* or other) algorithm is applied to each group of related experiments *i* (*i*=1,2,…,*r*) separately. The latter will generate a list of *r* different clustering solutions, one per each group. The result is that *K* (*K*=*k*_1_+…+*k*_*r*_) different clusters are produced by the different groups. This clustering solution is disjoint in terms of the gene expression profiles produced in the different experiments. However, it is not disjoint in terms of the different participating genes, *i.e.* there will be genes which will belong to more than one cluster.

#### FCA-based analysis step

As discussed above, the *N* studied genes are grouped during the *Clustering step* into *K* clusters that are not guaranteed to be disjoint. This overlapping partition is further analysed and refined into a disjoint one by applying FCA. As mentioned above FCA is a principled way of automatically deriving a hierarchical conceptual structure from a collection of objects and their properties. The approach takes as input a matrix (referred to as the formal context) specifying a set of objects and the properties thereof, called attributes. In our case, a formal context consists of the set *G* of the *N* studied genes (objects), the set of clusters *C*=*C*_1_,*C*_2_,…,*C*_*K*_ produced by the clustering step (attributes), and an indication of which genes belong to which clusters. Thus, the context is described as a matrix, with the genes corresponding to the rows and the clusters corresponding to the columns of the matrix, and a value of 1 in cell *(i, j)* whenever gene *i* belongs to cluster *C*_*j*_. Subsequently, a formal concept for this context is defined to be a pair *(X, Y)* such that 

• *X*⊆*G* & *Y*⊆*C* & every gene in *X* belongs to every cluster in *Y*

• for every gene in *G* that is not in *X*, there is a cluster in *Y* that does not contain that gene

• for every cluster in *C* that is not in *Y*, there is a gene in *X* that does not belong to that cluster.

The family of these concepts obeys the mathematical axioms defining a *concept lattice*. The constructed *lattice* consists of concepts where each one represents a subset of genes belonging to a number of clusters. The set of all concepts partitions the genes into a set of disjoint clusters.

## Validation setup

### Microarray datasets

The aforementioned consensus clustering algorithms are validated and compared on benchmark datasets where the true clustering is known. These datasets are composed by gene expression time series data obtained from a study examining the global cell-cycle control of gene expression in fission yeast *Schizosaccharomyces pombe*[1]. The study includes eight independent time-course experiments synchronized respectively by: 

1. elutriation: three independent biological repeats (*elu1*, *elu2*, *elu3*);

2. cdc25 block-release: two independent biological repeats, of which one in two dye-swapped technical replicates (*cdc25-1*, *cdc25-2.1*, *cdc25-2.2*) and in addition, one experiment in a sep1 mutant background (*cdc25-sep1*);

3. a combination of both methods: elutriation and cdc25 block-release (*elu-cdc10*) as well as elutriation and cdc10 block-release (*elu-cdc25*).

Thus, nine different expression test sets are available. In the pre-processing phase the rows with more than 25% missing entries are filtered out from each expression matrix and any other missing expression entries are imputed by the DTWimpute algorithm presented in [34]. In this way nine complete matrices are obtained.

The authors in [1] identified 407 genes as cell-cycle regulated subjected to clustering which resulted in the formation of 4 separate clusters. Subsequently, the time expression profiles of these genes are extracted from the complete data matrices and thus nine new matrices are constructed. Note that some of these 407 genes are removed from the original matrices during the pre-processing phase, *i.e.* each dataset may have a different set of genes. Thus a set of 376 different genes are present in the nine pre-processed datasets in total.

Two different benchmark datasets are constructed from the original nine pre-processed matrices and used in the validation process: 

1. The genes that are not present in the intersection of the nine pre-processed datasets are removed. The latter produces a subset of 267 genes. Subsequently, the time expression profiles of these genes are extracted from the complete data matrices and thus nine new matrices which form our **test corpus 1** are constructed.

2. The initial complete datasets are divided into three groups with respect to the used synchronization method. The overlapping genes within each group are as follows: a subset of 286 common genes in the elutriation datasets, a subset of 350 common genes in the cdc25 block-release datasets and a subset of 374 common genes in the datasets synchronized by the combination of both methods. For each of the three groups only these common genes are retained. As a result of this nine new matrices which form our **test corpus 2** are built. Notice that the nine different dataset contain 374 different genes in total.

The benchmark datasets are normalized by applying a data transformation method as proposed in [35].

### Consensus clustering algorithms

In order to validate the proposed general *FCA-enhanced* approach, we applied two different consensus clustering methods: 1) an algorithm integrating multiple partitioning results and 2) a *PSO-based* clustering method.

These two consensus clustering methods approach in a different way the initialization of the cluster centres and the production of the final clustering partition. Both methods initially restrict the studied genes for each group to those contained in all datasets of the group.

The first algorithm, referred to as *Integrative*, initializes the cluster centres for each group of experiments using the information contained in the datasets of the group in an integrated manner. This step is performed using a matrix constructed by concatenating the expression matrices in each group. The k-means algorithm is then applied to each expression matrix in the group to generate a set of partition matrices for each group. Utilizing information on the quality of the microarrays, weights are assigned to the experiments and are further used in the integration process in order to obtain more realistic overall partition for each group. The data transformation method proposed in [35] is used to evaluate the quality of the considered microarrays. It is applied to each expression matrix and the number of standardized genes is considered as a quality measure for this matrix. This step results in the assignment of a weight to each expression matrix in the group, *i.e.* the different experiments in the group will contribute to the final partitioning result to a different extent. A detailed explanation of the *Integrative* consensus clustering algorithm can be found in [13].

The second consensus clustering method, referred to as *PSO-based*, employs a PSO approach to cluster gene expression data across multiple experiments. Each experiment (dataset) defines a particle which is initialized with a set of *k* cluster centroids obtained after performing the k-means clustering algorithm applied over the experiment. The final (optimal) clustering solution for each group of experiments is found by updating the particles using the information on the best clustering solution obtained by each experiment and the entire set of experiments in the group. A detailed explanation of the *PSO-based* consensus clustering algorithm can be found in [14].

### Cluster validation measures

One of the most important issues in cluster analysis is the validation of the clustering results. Essentially, cluster validation techniques are designed to find the partitioning that best fits the underlying data, and should therefore be regarded as a key tool in the interpretation of the clustering results. Since none of the clustering algorithms performs uniformly well under all scenarios, it is not reliable to use a single cluster validation measure, but instead to use at least two that reflect different aspects of a partitioning. In this sense, we have implemented two different validation measures for estimating the quality of the clusters: 

1. Connectivity: for assessing connectedness;

2. Silhouette Index (SI): for assessing compactness and separation properties of a partitioning.

#### Connectivity

The Connectivity captures the degree to which genes are connected within a cluster by keeping track of whether the neighbouring genes are put into the same cluster [36]. Let us define *m*_*i*(*j*)_ as the *j*-th nearest neighbour of gene *i*, and let $\chi_{im_{i(j)}}$ be zero if *i* and *j* are in the same cluster and 1/*j* otherwise. Then for a particular clustering solution *C*_1_,*C*_2_,…,*C*_*k*_ of matrix *M*, which contains the expression values of *m* genes (rows) in *n* different experimental conditions or time points (columns), the connectivity is defined as

$$\text{Conn}(c)=\overset{m}{\underset{i=1}{\sum }}\overset{n}{\underset{j=1}{\sum }}\chi_{im_{i(\;j)}}.$$

The Connectivity has a value between *zero* and *infinity* and should be *minimized*.

#### Silhouette index

The Silhouette Index (SI) reflects the compactness and separation of clusters [37]. Suppose *C*_1_,*C*_2_,…,*C*_*k*_ is a clustering solution (partition) of matrix *M*, which contains the expression profiles of *m* genes. Then, the SI is defined as

$$s(k)=\frac{1}{m}\overset{m}{\underset{i=1}{\sum }}\left(b_{i}-a_{i}\right)/\max\left\{a_{i},b_{i}\right\},$$

where *a*_*i*_ represents the average distance of gene *i* to the other genes of the cluster to which the gene is assigned, and *b*_*i*_ represents the minimum of the average distances of gene *i* to genes of the other clusters.

The values of the Silhouette Index vary from *-1* to *1* and a *higher value* indicates better clustering results.

## Results and discussion

The validation process presented below has two main goals: 1) to illustrate that different clustering methods usually generate gene partitions on expression data, which may exhibit considerable inconsistencies and discrepancies in terms of clustering quality and gene composition 2) to demonstrate the added value of an *FCA-enhanced* clustering approach for overcoming and diminishing such differences and preserving relevant biological signals. For this purpose, the following partitions have been generated and compared: 

1. two consensus partitions over **test corpus 1** using respectively the original versions of the *Integrative* and the *PSO-based* consensus clustering algorithms;

2. two times three consensus partitions, one for each group of experiments from **test corpus 2**, using respectively the grouped versions of the *Integrative* and the *PSO-based* consensus clustering algorithms as specified in the foregoing section;

3. two concept lattices derived by applying FCA on the two sets of group partitions (see above) produced respectively from the grouped versions of the *Integrative* and the *PSO-based* methods.

### Clustering performance

In this section, we evaluate and compare the clustering performance of the two consensus clustering algorithms discussed in the foregoing section on the benchmark datasets described above by using two cluster validation measures: Silhouette Index and Connectivity.

**Figure 2:**
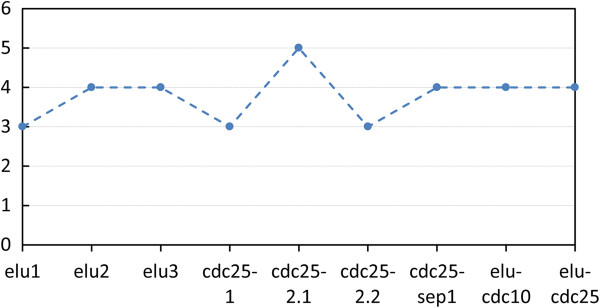
**Optimal number of clusters as determined using the Connectivity validation index for the 9 different experiments.** For 5 of the 9 datasets *k*=4 is identified as the optimal cluster number.

The partitioning algorithms such as k-means contain the number of clusters (*k*) as a parameter and their major drawback is the lack of prior knowledge for that number. Therefore, we initially identified the number of cluster centres by running the k-means clustering algorithm on each dataset for values of *k* between 2 and 10 [32], [33]. Subsequently, the quality of the obtained clustering solutions is assessed using the Connectivity validation index. We focused on the values of *k* at which a significant local change in value of the index occurs [32]. The optimal number of clusters for the different experiments range between 3 and 5 as can be seen in Figure 2. However, *k*=4 prevails (encountered in five matrices) and therefore it is used for our experiments.

**Figure 3:**
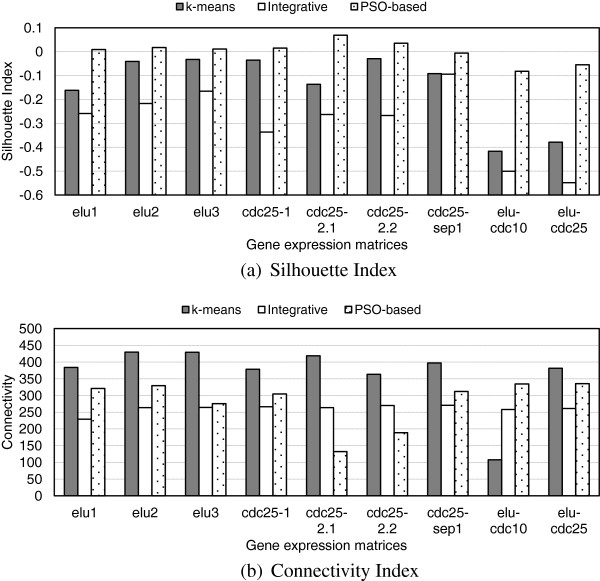
**Comparison of the SI (a) and Connectivity (b) values generated by the standard k-means algorithm, and the** ***Integrative* and the** ***PSO-based* consensus clustering algorithms on test corpus 1.**

Figure 3 compares for **test corpus 1**, the SI and Connectivity values corresponding to the partitions generated by the standard k-means on each individual matrix versus the values calculated on the individual matrices using the overall consensus partitions produced respectively by the *Integrative* and the *PSO-based* consensus clustering algorithm. One can observe that the SI scores obtained by the *PSO-based* clustering technique are better than those generated by the *Integrative* clustering and k-means for all the individual matrices. However, under the Connectivity measure, the *Integrative* solution exhibits better performance than the other algorithms (in 7 of the 9 experiments for the *PSO-based* clustering algorithm and in 8 of the 9 for k-means). The obtained results are explained by the fact that the *PSO-based* clustering algorithm favours the production of compact and well separated clusters instead of well connected, since it finds the final clustering solution by updating the cluster centres using the information on the best clustering solution generated by each experiment and the entire set of experiments. The *Integrative* clustering algorithm on the other hand has a bias towards well connected clusters as it produces the lowest SI scores in comparison to k-means and the *PSO-based* algorithms. Clearly, each clustering algorithm may introduce biases due to its specific characteristics. Therefore a generic solution, such as supported by the FCA approach, that diminishes the differences and preserves relevant biological signals is required to be applied for further data analysis.

Next, the performance of the grouped versions of the *Integrative* and *PSO-based* consensus clustering algorithms on **test corpus 2** is considered. Initially, the nine datasets are divided into three groups with respect to the used synchronized method: 

1. elutriation datasets: *elu1*, *elu2*, *elu3*;

2. cdc25 block-release datasets: *cdc25-1*, *cdc25-2.1*, *cdc25-2.2*, *cdc25-sep1*;

3. datasets synchronized by the combination of both methods: *elu-cdc10*, *elu-cdc25*.

Afterwards, the number of cluster centres is identified for each group using the Connectivity measure. The selected optimal number of clusters for the three groups of experiments is as follows: elutriation datasets: *k*=4; cdc25 block-release datasets: *k*=6, and the combined ones: *k*=5. As a result 15 different clusters (elutriation: clusters 0-3, cdc25 block-release: clusters 4-9 and combination of both: clusters 10-14) in total are produced by each of the two consensus clustering methods.

**Figure 4:**
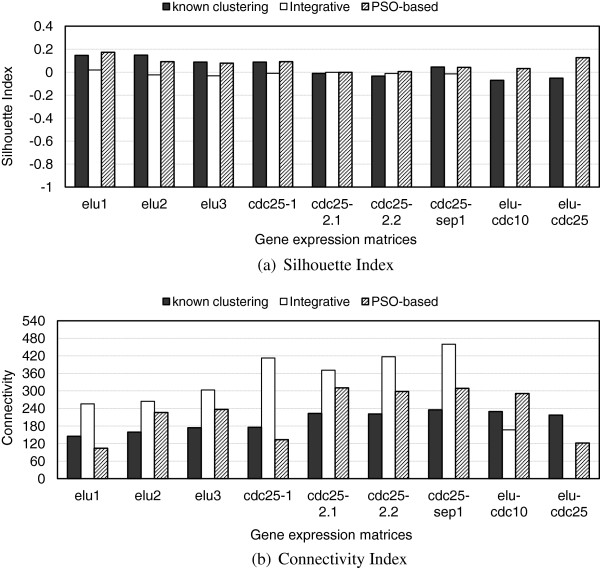
**Comparison of the SI (a) and Connectivity (b) values generated by the known clustering solution published in [**[1]**], and those obtained by applying the grouped-versions of the** ***Integrative* and** ***PSO-based* consensus clustering algorithms on test corpus 2.**

Figure 4 compares the SI and Connectivity values calculated on the individual matrices using the partitions produced by the known clustering solution published in [1], and those generated by the grouped *Integrative* and *PSO-based* clustering solutions. According to the SI indices in Figure 4(a), the *PSO-based* solution clearly outperforms the *Integrative* one. It also exhibits better performance than the *Integrative* clustering solution in 7 of the 9 experiments under the Connectivity validation measure. In addition, the *PSO-based* clustering solution is better than the known one in 6 of the 9 experiments under the SI validation index and respectively, in 3 of the 9 experiments under the Connectivity index. These results are further analyzed in the next section.

### Cluster consistency

In this section, we analyse the impact of the different clustering methods on the final gene partition. The degree of pairwise overlap between the different gene clusters generated by the *Integrative* and the *PSO-based* consensus clustering algorithms is calculated and compared. Assume that we have a gene cluster *c*_*i*_ produced by the *Integrative* algorithm and a gene cluster *c*_*j*_ coming from the *PSO-based* clustering. Then the degree of overlap is calculated as follows:

$$d_{\text{ij}}=\frac{\#\left(c_{i}\cap c_{j}\right)}{\max\left(\#c_{i},\#c_{j}\right)}100.$$

In this way, *d*_*i* *j*_ will be equal to 100 in case of full overlap between the clusters *i* and *j*, 0 in case of no overlap and between 0 and 100 otherwise.

**Figure 5:**
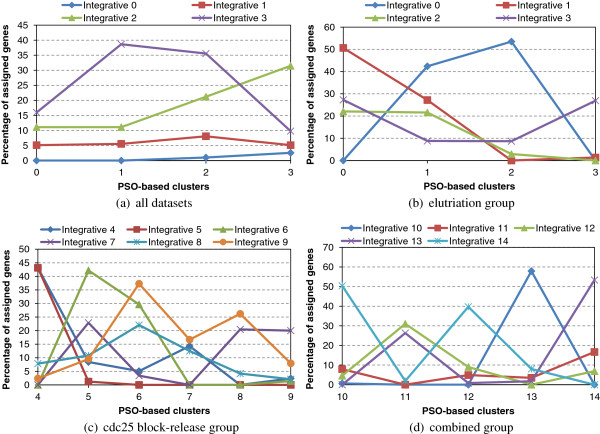
**Consensus clustering assignment overlap:*Integrative* vs.***PSO-based*. Figure **(a)** compares the consensus gene partitions generated by the consensus clustering algorithms on **test corpus 1** while Figures **(b)**, **(c)**, **(d)** depict the overlap between the consensus clustering assignment of the clustering algorithms on **test corpus 2** for each group of experiments separately.

The consensus gene partitions generated respectively by the original *Integrative* and the *PSO-based* consensus clustering algorithms on **test corpus 1** are compared in Figure 5(a). The best overlap is recorded for the pairs: (PSO 1, Integrative 3), (PSO 2, Integrative 3), (PSO 2, Integrative 2) and (PSO 3, Integrative 2). Evidently, the genes of PSO cluster 0 are almost uniformly distributed between Integrative 1, Integrative 2 and Integrative 3, those of PSO 1 are allocated to Integrative 3, the genes of PSO 2 are mainly distributed between Integrative 2 and Integrative 3 and PSO 3 has a high overlap with Integrative 2.

Figures 5(b), (c), (d) depict the overlap between the consensus clustering assignment of *PSO-based* versus the *Integrative* clustering algorithms on **test corpus 2** for each group of experiments separately (respectively elutriation, cdc25 block-release and the combined). The best overlap is recorded for the following pairs: 

• elutriation: (PSO 0, Integrative 1), (PSO 2, Integrative 0), (PSO 3, Integrative 3),

• cdc25 block-release: (PSO 4, Integrative 5), (PSO 4, Integrative 4), (PSO 5, Integrative 6), (PSO 6, Integrative 9), (PSO 8, Integrative 9), (PSO 9, Integrative 7),

• combined: (PSO 10, Integrative 14), (PSO 12, Integrative 14), (PSO 13, Integrative 10) and (PSO 14, Integrative 13).

The high degree of pairwise overlap between the different gene clusters generated by the *Integrative* and the *PSO-based* consensus clustering algorithms suggests that there is a certain consistency in the resulting clustering solutions. The next section elaborates on this effect by applying FCA on both consensus clustering solutions consolidating the different groups of experiments.

### Results from the FCA-enhanced Step

The gene partitions produced by the grouped versions of the *Integrative* and *PSO-based* consensus clustering algorithms on **test corpus 2** are further analysed by applying FCA.

**Figure 6:**
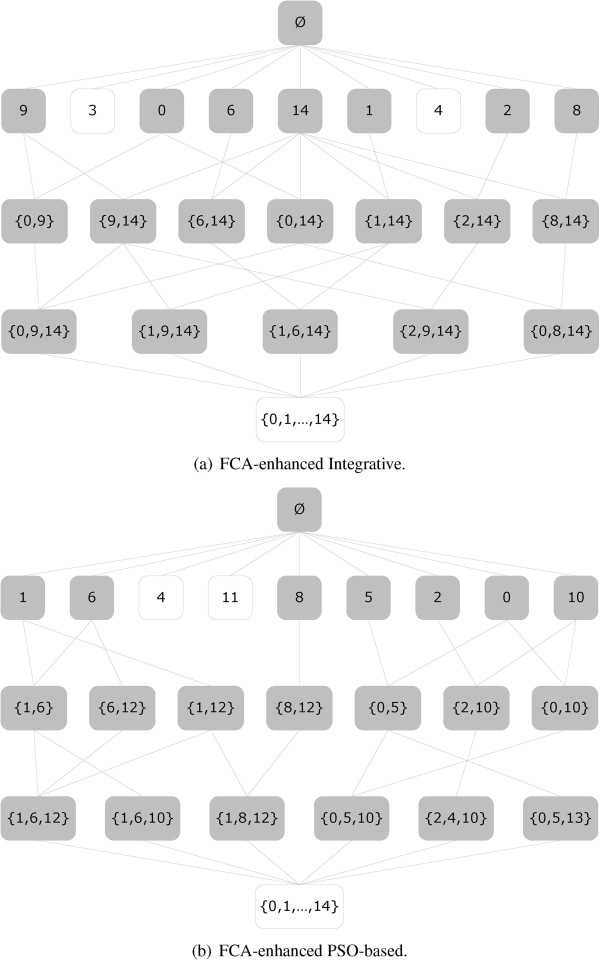
**The concept lattices generated by the** ***FCA-enhanced* consensus clustering produced by the** ***Integrative* (a) and the** ***PSO-based* (b) clustering algorithms respectively.**

First, we create a context that consists of the set of 374 studied genes and the set of 15 clusters produced by the grouped version of the *Integrative* consensus clustering algorithm. It produces a lattice of 133 concepts for this context (see Figure 6(a)). However, 74 concepts have support 0, *i.e.* the benchmark genes are grouped into 59 disjoint clusters (concepts). Further a context that consists of the set of 374 studied genes and the set of 15 clusters produced by the grouped version of the *PSO-based* clustering algorithm is built. Subsequently, a lattice of 109 concepts for this context is generated (see Figure 6(b)). The FCA step partitions the benchmark gene set in 85 disjoint clusters (concepts) in total since the rest of the concepts have support 0. It is interesting to notice that all the concepts consisting of three clusters in both disjoint partitions (21 such concepts exist in the *Integrative* partition and 29 in the *PSO-based*) contain clusters produced by each of the three groups of experiments (elutriation, cdc25 block-release, combined).

**Figure 7:**
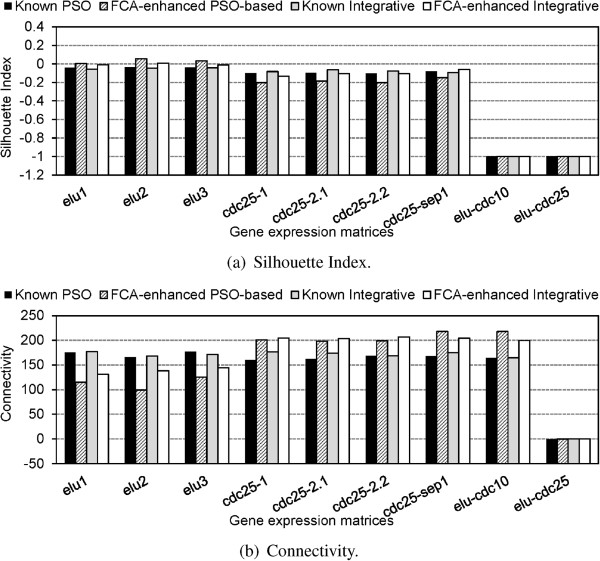
**Comparison of the SI (a) and Connectivity (b) values for the expression matrices formed by the genes contained in the concepts from Table**1**.**

Let us consider the concepts, each consisting of three clusters, with support above 0.03 for both *FCA-enhanced* clustering partitions in Table 1. The SI and Connectivity values for the expression matrices formed by the genes contained in these concepts are compared to the known clustering solution published in [1]. This was executed by extracting the known clustering solutions for the PSO and Integrative concepts in Table 1. The results from Figure 7 show that in contrast to the results obtained for the clustering results before the FCA step (see Figures 3 and 4), the SI and Connectivity performances of the two clustering algorithms follow a similar trend. Thus, it appears that FCA step is able to overcome cluster assignment differences and performance discrepancies mostly attributed to the specificities of the different clustering methods. Additionally, for the *elutriation* experiments FCA has better SI and Connectivity than the known clustering solution for both PSO and Integrative. However, for *cdc25 block-release* the FCA performs for both measures a bit worse than the known solutions.

**Table 1:** All concepts consisting of threeclusters with support above 0.03

**PSO-based**	**Integrative**
{1, 6, 10}	{0, 9, 14}
{0, 5, 13}	{1, 6, 14}
{1, 6, 12}	{0, 8, 14}
{2, 4, 10}	{2, 9, 14}
{0, 5, 10}	{1, 9, 14}
{1, 8, 12}	

Let us investigate whether some correspondence exists between the *PSO-based* and the *Integrative* concepts in Table 1. For this purpose we consider the overlapping pairs of clusters of the grouped FCA partitions identified in the foregoing section (Figures 5(b), (c), (d)). Table 2 presents the correspondence between the *PSO-based* and the *Integrative* concepts in Table 1 in terms of the percentage minimum cluster assignment overlap as extracted from the results in Figures 5(b), (c), (d). Note that Table 2 does not include concepts, which exhibit significantly low overlap (lower than 20%) with any other concept in Table 1. Next, each of the 9 different concepts in Table 2 are subjected to analysis with the BiNGO tool [38], in order to determine which Gene Ontology categories are statistically overrepresented in each concept. The results are generated for a cut-off p-value of 0.05 and Benjamini and Hochberg (False Discovery Rate) multiple testing correction. For each gene concept a table is generated consisting of five columns: (1) the GO category identification (GO-id); (2) the multiple testing corrected p-value (p-value); (3) the total number of genes annotated to that GO term divided by total number of genes in the test set (cluster frequency); (4) the number of selected genes versus the total GO number (total frequency); and (5) a detailed description of the selected GO categories (description).

**Table 2:** **Percentage minimum overlap between the concepts from Table**1

**PSO-based integrative**	**{1, 6, 10}**	**{1, 6, 12}**	**{0, 5, 10}**	**{1, 8, 12}**
{0, 9, 14}	35*%*	35*%*	-	25*%*
{1, 6, 14}	20*%*	20*%*	40*%*	-
{0, 8, 14}	20*%*	20*%*	-	-
{2, 9, 14}	20*%*	20*%*	-	20*%*
{1, 9, 14}	25*%*	25*%*	-	25*%*

Only 6 of the 9 FCA concepts are assigned GO categories by the BiNGO tool: 

• Integrative-{0, 9, 14} contains 41 genes annotated to 26 GO categories (25 have total frequency >0.0*%*), most of which refer to the **regulation of protein kinase activity** or **regulation of cell-cycle process** or **regulation of metabolic process**;

• Integrative-{0, 8, 14} contains 22 genes annotated to 25 GO categories (22 have total frequency >0.0*%* and cluster frequency >10.0*%*), dominated by **sister chromatid segregation** and **beta-glucan process regulation categories**;

• Integrative-{1, 6, 14} contains 21 genes annotated to 21 GO categories (15 have total frequency >0.0*%*), most of which refer to **regulation of mRNA stability**;

• PSO-{1, 6, 10} contains 29 genes connected with 19 GO categories (10 have total frequency >0.0*%*), most of which refer to **cell-cycle control** or **regulation of DNA replication** or **sister chromatid segregation**;

• PSO-{1, 6, 12} contains 18 genes connected with 5 GO categories (only 3 have total frequency >0.0*%*), all referring to the regulation of **sister chromatid cohesion and segregation**;

• PSO-{1, 8, 12} contains 12 genes annotated to 22 GO categories (16 have total frequency >0.0*%*) dominated by RNA **metabolic processing** related categories.

It can be observed that the correspondences between the *PSO-based* and *Integrative* concepts presented in Table 2 are also supported by the above GO categories e.g. sister chromatid segregation, DNA and cell-cycle regulation, metabolic processing, etc. These correspondences suggest that although both algorithms optimize different clustering characteristics (SI and Connectivity indices in Figure 4), FCA is able to construct similar consensus clustering lattices that are representative for all the datasets. Evidently, the proposed FCA-enhanced approach is a generic consensus clustering technique that is not dependent on the applied clustering algorithm. This means that one can use a customized algorithm suited for the specific characteristic of each dataset group and consolidate the resulting clustering solutions of the involved groups by using FCA.

## Conclusions

In this paper we introduced a novel consensus clustering technique which proposes a general approach to the combination of clustering algorithms with *Formal Concept Analysis (FCA)* for deriving representative clustering solutions from multiple gene expression matrices. This approach involves three distinctive steps: (i) the studied microarray experiments are partitioned into groups of related datasets with respect to a predefined criterion, (ii) a consensus clustering algorithm is applied to each group of experiments separately, (iii) the clustering solutions produced by the different groups are pooled together and further analyzed by employing FCA. The performance of the proposed consensus clustering algorithm is evaluated on a test set of nine time series expression datasets obtained from a study examining the global cell-cycle control of gene expression in fission yeast *Schizosaccharomyces pombe*. In addition, in step (ii) of the proposed approach two different consensus clustering algorithms (*Integrative* and *PSO-based*) are applied for the validation process. The presented experimental results demonstrate that the proposed *FCA-enhanced* clustering algorithm is a robust data integration technique able to produce good quality clustering solution that is representative for the whole set of experiments. In addition, the employment of FCA allows performing a subsequent data analysis, which provides useful insights on the biological role of genes contained in the same FCA concepts. Our future work will focus on further exhaustive analysis of the composition and relationships between the different FCA concepts. Moreover, our longterm aim is to further evaluate the generalisability of *FCA-enhanced* consensus clustering technique by conducting experiments with other clustering algorithms and microarray datasets.

## Implementation and availability

The used cluster validation measures and the *Integrative* consensus clustering algorithm have been implemented in C++. In addition, the *PSO-based* clustering algorithm has been implemented in Java. The publicly available open source machine learning software WEKA [39] is used by this implementation for the particle initialization and for the gene assignment to the different clusters. Finally, FCA is performed by using publicly available tools [40].

## Endnotes

^a^ The number of clusters *k*, is initially identified by analyzing the quality of the obtained clustering solutions generated on the involved datasets for a range of different numbers of clusters.

^b^ The velocity vectors are initialized by zeros.

## Competing interests

The authors declare that they have no competing interests.

## Authors’ contributions

ET conceived the project. AH, VB and ET designed the methodology and experiments. AH performed the experiments. AH, VB and ET interpreted the results and drafted the manuscript. All authors have read and approved the final manuscript.

## References

[B1] Rustici G Mata J Kivinen K Lió P Penkett CJ Burns G Hayles J Brazma A Nurse P Bähler J **Periodic gene expression program of the fission yeast cell cycle** Nat Genet 2004 36 8 809 817 10.1038/ng1377 15195092

[B2] Alizadeh AA Eisen MB Davis RE Ma C Lossos IS Rosenwald A Boldrick JC Sabet H Tran T Yu X Powell JI Yang L Marti GE Moore T Hudson JJr Lu L Lewis DB Tibshirani R Sherlock G Chan WC Greiner TC Weisenburger DD Armitage JO Warnke R Levy R Wilson W Grever MR Byrd JC Botstein D Brown PO **Distinct types of diffuse large b-cell lymphoma identified by gene expression profiling** Nature 2000 403 6769 503 511 10.1038/35000501 10676951

[B3] Golub TR Slonim DK Tamayo P Huard C Gaasenbeek M Mesirov JP Coller H Loh ML Downing JR Caligiuri MA Bloomfield CD Lander ES **Molecular classification of cancer: class discovery and class prediction by gene expression monitoring** Science 1999 286 5439 531 537 10.1126/science.286.5439.531 10521349

[B4] Gilks WR Tom BDM Brazma A **Fusing microarray experiments with multivariate regression** Bioinformatics 2005 21 suppl 2 137 143 16204093 10.1093/bioinformatics/bti1123

[B5] Choi JK Yu U Kim S Yoo OJ **Combining multiple microarray studies and modeling interstudy variation** Bioinformatics 2003 19 suppl 1 84 90 10.1093/bioinformatics/btg1010 12855442

[B6] Zhou XJ Kao MCJ Huang H Wong A Nunez-Iglesias J Primig M Aparicio OM Finch CE Morgan TE Wong WH **Functional annotation and network reconstruction through cross-platform integration of microarray data** Nat Biotechnol 2005 23 2 238 243 10.1038/nbt1058 15654329

[B7] Garrett-Mayer E Parmigiani G Zhong X Cope L Gabrielson E **Cross-study validation and combined analysis of gene expression microarray data** Biostatistics 2008 9 2 333 354 17873151 10.1093/biostatistics/kxm033

[B8] Filkov V Skiena S **Integrating microarray data by consensus clustering** Int J Artif Intell Tools 2004 13 4 863 880 10.1142/S0218213004001867

[B9] Johnson E Kargupta H **Collective, hierarchical clustering from distributed, heterogeneous data** Large-Scale Parallel KDD Syst 1999 1759 221 244

[B10] Topchy A Jain AK Punch W **Clustering ensembles: models of consensus and weak partitions** IEEE Trans Pattern Anal Mach Intell 2005 27 12 1866 1881 16355656 10.1109/TPAMI.2005.237

[B11] Strehl A Ghosh J **Cluster ensembles—a knowledge reuse framework for combining multiple partitions** J Mach Learn Res 2003 3 583 617

[B12] Dempster AP Laird NM Rubin DB **Maximum likelihood from incomplete data via the em algorithm** J Roy Stat Soc B 1977 39 1 1 38

[B13] Kostadinova E Boeva V Lavesson N **Clustering of multiple microarray experiments using information integration** Inf Technol Bio-and Med Inform 2011 6865 123 137 10.1007/978-3-642-23208-4_12

[B14] Boeva V Hristoskova A Tsiporkova E **Clustering of multiple dna microarrays through combination of particle swarm intelligence and k-means**6th International Conference on Computational Intelligence and Bioinformatics: Modelling, Identification, and Simulation 2011 Pittsburgh, USA: ACTA Press 32 38

[B15] Hristoskova A Boeva V Tsiporkova E **An integrative clustering approach combining particle swarm optimization and formal concept analysis** Proceedings of Information Technology in Bio-and Medical Informatics 2012 Vienna, Austria: Springer Berlin Heidelberg 84 98

[B16] MacQueen J **Some methods for classification and analysis of multivariate observations** Proceedings of the Fifth Berkeley Symposium on Mathematical Statistics and Probability, vol. 1: 21 June - 18 July, 1965 and 27 December, 1965 - 7 January, 1966; California, USA 1967 Berkeley, Calif.: University of California Press 281 297

[B17] Kaufman L Rousseeuw PJ **Fitting groups in data: an introduction to cluster analysis** J Am Stat Ass 1991 86 415 830 832

[B18] Kennedy J Eberhart R **Particle swarm optimization** IEEE International Conference on Neural Networks, vol. 4 1995 IEEE 1942 1948

[B19] Shi Y Eberhart R **A modified particle swarm optimizer** IEEE International Conference on Evolutionary Computation 1998 IEEE 69 73

[B20] Omran M Engelbrecht AP Salman A **Particle swarm optimization method for image clustering** Int J Pattern Recogn Artif Intell 2005 19 3 297 322 10.1142/S0218001405004083

[B21] Omran M Salman A Engelbrecht A **Image classification using particle swarm optimization** Proceedings of the 4th Asia-Pacific Conference on Simulated Evolution and Learning, vol. 1: 18-22 November 2002; Orchid Country Club, Singapore 2002 Singapore: [Nanyang Technological University, School of Electrical & Electronic Engineering] 370 374

[B22] Van der Merwe D Engelbrecht A **Data clustering using particle swarm optimization** IEEE Congress on Evolutionary Computation, vol. 1 2003 Canberra, Australia: IEEE 215 220

[B23] Xiao X Dow ER Eberhart R Miled ZB Oppelt RJ **Gene clustering using self-organizing maps and particle swarm optimization**17th International Symposium on Parallel and Distributed Processing 2003 Nice, France: IEEE 10 21

[B24] Kuo RJ Syu YJ Chen Z-Y Tien FC **Integration of particle swarm optimization and genetic algorithm for dynamic clustering** Inform Sci 2012 195 124 140

[B25] Ganter B Stumme G Wille R Formal Concept Analysis: Foundations and Applications, vol. 3626 2005 Berlin, Heidelberg: Springer

[B26] Besson J Robardet C Boulicaut JF **Constraint-based mining of formal concepts in transactional data** Adv Knowl Discov Data Mining 2004 3056 615 624

[B27] Besson J Robardet C Boulicaut JF Rome S **Constraint-based concept mining and its application to microarray data analysis** Intell Data Anal 2005 9 1 59 82

[B28] Choi V Huang Y Lam V Potter D Laubenbacher R Duca K **Using formal concept analysis for microarray data comparison** J Bioinform Comput Biol 2008 6 1 65 10.1142/S021972000800328X 18324746

[B29] Potter DP **A combinatorial approach to scientific exploration of gene expression data: an integrative method using formal concept analysis for the comparative analysis of microarray data** PhD thesis. Citeseer; 2005

[B30] Kaytoue-Uberall M Duplessis S Napoli A **Using formal concept analysis for the extraction of groups of co-expressed genes** Model Computat Optimization Inf Syst Manage Sci 2008 14 445 455

[B31] Kaytoue M Kuznetsov S Napoli A Duplessis S **Mining gene expression data with pattern structures in formal concept analysis** Inf Sci 2011 181 10 1989 2001 10.1016/j.ins.2010.07.007

[B32] Halkidi M Batistakis Y Vazirgiannis M **On clustering validation techniques** J Intell Inform Syst 2001 17 2 107 145

[B33] Theodoridis S Koutroubas K Pattern Recognition 1999 New York: Academic Press

[B34] Tsiporkova E Boeva V **Two-pass imputation algorithm for missing value estimation in gene expression time series** J Bioinform Comput Biol 2007 5 5 1005 1022 10.1142/S0219720007003053 17933008

[B35] Boeva V Tsiporkova E **A multi-purpose time series data standardization method** Intell Syst Theory Pract 2010 299 445 460 10.1007/978-3-642-13428-9_22

[B36] Handl J Knowles J Kell DB **Computational cluster validation in post-genomic data analysis** Bioinformatics 2005 21 15 3201 3212 10.1093/bioinformatics/bti517 15914541

[B37] Rousseeuw PJ **Silhouettes: a graphical aid to the interpretation and validation of cluster analysis** J Comput Appl Math 1987 20 53 65

[B38] Maere S Heymans K Kuiper M **Bingo: a cytoscape plugin to assess overrepresentation of gene ontology categories in biological networks** Bioinformatics 2005 21 16 3448 3449 10.1093/bioinformatics/bti551 15972284

[B39] **Weka: data mining software in Java** [http://www.cs.waikato.ac.nz/ml/weka/]

[B40] **Galicia: Galois lattice interactive constructor** [http://www.iro.umontreal.ca/galicia/features.html]

